# Role of nurses in managing menstrual health using mobile tracking

**DOI:** 10.1097/01.NURSE.0000795280.98049.92

**Published:** 2021-10-22

**Authors:** Barbara N. Sanchez, Carl M. Maresh

**Affiliations:** **Barbara N. Sanchez** is a graduate teaching associate and research fellow in the Health & Exercise Science Program at The Ohio State University, where **Carl M. Maresh** is a professor and the chair of the Department of Human Sciences.

**Keywords:** menstrual cycle, reproductive health, technology, women's health

## Abstract

Mobile technologies provide a unique opportunity to improve menstrual health awareness, management, and care. Nurses should consider incorporating a mobile tracking component into patient-care settings to promote menstrual health awareness and advocacy and to help create individualized healthcare plans.

**Figure:**
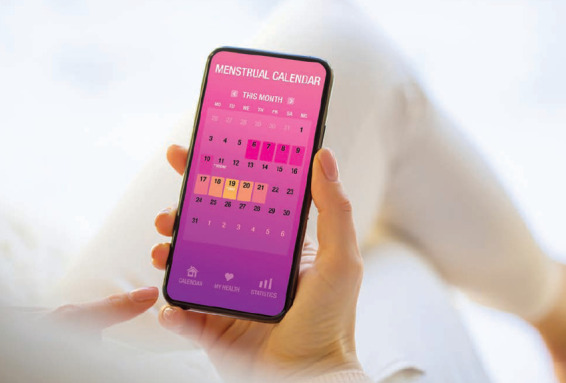
No caption available.

THE USE OF smartphones and other mobile technologies for tracking and managing behaviors has become increasingly widespread. Within the context of health and wellness, nearly 70% of adults in the US report tracking and/or self-monitoring a health condition or behavior.^1^ Furthermore, the use of mobile applications (hereafter referred to as “app” or “apps”) to track menstrual cycles is the fourth most popular health monitoring app among adults, and the second most popular among adolescents who menstruate.^2^ The inherent inter- and intra-individual variabilities of the menstrual cycle provide an opportunity for mobile apps to support an individualized approach to menstrual health awareness, management, and care.

In 2018, Starling, et al., surveyed 1,000 self-identified women through online channels and reported that 23.1% had or currently used an app to track their menstrual cycle, while 76.9% reported their intention to use a menstrual tracking app in the future.^3^ In a different survey that asked participants to choose between the use of a mobile app or a paper diary to log and track menstrual bleeding, 80% of participants preferred the app.^4^ These results suggest a growing interest in using a mobile app to track menstrual health, especially among younger women whose day-to-day activities involve technology and mobile device use.

Nurses are in a unique position to promote the use of mobile tracking apps for patients to monitor their menstrual cycles as part of delivering holistic care. Promoting the habit of tracking menstrual cycles allows engagement with patients on important topics such as menstrual health characteristics (such as cycle length and amount of flow) and navigating privacy concerns with menstrual tracking apps. Addressing these topics can help educate patients so they can monitor their health while keeping their private health information safe.

It is common for nurses to assess a patient's vital signs as part of a routine physical assessment. The American College of Obstetricians and Gynecologists (ACOG) released a committee opinion piece advocating for evaluation of menstrual cycle length and pattern of menses to be included as part of assessing vital signs within the Review of Systems (ROS) and History of Present Illness.^5^ Although clinical practices should not be solely based on expert opinion, those in patient-care settings need to balance evidence-based practices with their clinical experiences to identify novel ways to enhance menstrual care. This inclusion of menstrual health evaluation in the ROS can emphasize the importance of menstrual health in the overall health and wellness assessment and care when coupled with evidence-based approaches and comprehensive patient education.

## Why individuals track their menstrual cycles

Tracking variables associated with menstrual health is an important way to monitor one's health status and improve physical and mental wellbeing. The reasons and motivations for routinely logging and tracking menstrual cycles include gaining an increased awareness of bodily functions and monitoring changes throughout the cycle, preparing for menses and social activities, conceiving, and having more informed conversations with healthcare providers.^6^,^7^ Thus, it appears that individuals who menstruate want to understand their body functions and validate their experience throughout their cycle. For example, changes to the menstrual cycle, such as excessive bleeding compared with months prior, can indicate that something in the body is out of balance. By understanding what is normal or not, an individual is in a better position to manage her acute and long-term health.

Menstruation-related experiences may share commonalities, but many experiences remain unique to the individual. Menstrual-related symptoms can vary among individuals due to cyclic hormonal changes and interactions with extrinsic factors such as the environment, physical activity, stress, diet, and sleep. By tracking menstrual cycles, individuals can learn more about how they respond to these factors, which is an important step toward better self-management.

As an extension of current patient education practices, nurses can share information about menstrual health and initiate conversations about menstrual tracking strategies when presenting general health and wellness information to patients, especially for nurses in pediatrics, women's health practices, and primary care. To illustrate the strengths and limitations of mobile tracking apps, paper logs, and simple memorization as methods of tracking menstrual cycle characteristics, see *Strengths and limitations of menstrual health tracking strategies*. Sharing this valuable information can help patients learn more about themselves and their bodies and feel more comfortable discussing health-related concerns, which can improve the management of their care.

## Benefits of digital tracking

While menstruation can usually be prepared for, it can occur unexpectedly from time to time. It can occur earlier or later than expected, and not being effectively prepared for the event may lead to increased levels of stress and negative emotions towards one's self and body. Being prepared for menses may have social implications as well.^7^ Scheduling activities like group gatherings and vacations around menses can prevent physical and social discomfort during a time of planned relaxation and connection. Being aware of patterns prior to, during, and after menstrual events can empower individuals to be proactive about their menstrual-related care, and from a societal perspective, help combat menstrual stigma through individual knowledge and action.

Most tracking strategies allow the user to track multiple related variables. Documenting multiple variables can allow for connections to be made between menstrual events and physical, emotional, and mental signs and symptoms. As one survey participant shared, “I have learned so much about my body/cycle from using the app, like how certain symptoms tell me I'm about to start my period...I always get moody around this particular time every month, etc.”^6^ Greater emphasis on tracking menstrual cycles should be part of conversations about health and wellness. Within this context, nurses can be menstrual health advocates by serving as a source of information and support when working with patients who menstruate.

Preparing the body for pregnancy is the primary physiologic purpose for the menstrual cycle, thereby, tracking menstrual cycles can serve as an important tool for successful conception. Tracking can assist in identifying the fertile window more easily, increasing the chances of pregnancy. Additionally, tracking can be used to reduce the likelihood of getting pregnant. Individuals may also track their cycles to inform healthcare providers about their wellbeing and health-related concerns. Individuals must advocate for their health, as not all physical and mental signs and symptoms often associated with the menstrual cycle are physiologically normal. For example, menstrual cramps are common for many individuals, but having debilitating and painful cramps may not be normal and may be an indication of an underlying health problem. Providing collected data can help healthcare providers know more about a patient's health profile, which is useful for developing an individualized healthcare action plan.

**Table:** Strengths and limitations of menstrual health tracking strategies

Mobile App		Paper Log		Simple Memorization	
Strengths	Limitations	Strengths	Limitations	Strengths	Limitations
Ease of use Users can enter data in real time Users can track a wide range of variables across time Apps can store large amounts of data App feature can include menses and ovulation prediction	Apps need consistent and accurate user engagement Learning curve associated with app use (although most people can learn to use an app quickly^4^) Privacy & data safety concerns	Individuals can track few to multiple variables across time (depends on log content, length) Secured data (individual controls information access)	Individuals can track only a few variables across time (depends on log content, length) Manual Menses and Ovulation predictions Requires physical space for storage over time	Nothing to write down Nothing to store Individual chooses which variables to keep track of	Recall bias can negatively impact tracking Information can be forgotten Limited number of variables available to track across time Trends can be difficult to identify

Regardless of why individuals track their menstrual cycles, tracking is highly beneficial for self-management and health monitoring. It helps individuals identify what works and what does not work for their bodies, thereby contributing to greater self-awareness of one's health, which promotes successful health behavior change.^7^,^8^ Increased self-awareness can lead to greater control of health conditions as individuals can be more proactive about seeking appropriate care.^8^ Considering the economic strain of healthcare and its impact on healthcare systems, the more self-aware a person becomes, the greater their autonomy in taking charge of their health and wellness, thus helping reduce burdens on the healthcare system.^3^,^8^

## Accuracy of tracking apps for practical use and scientific investigations

The efficacy of a menstrual cycle tracking app depends on an appropriate interface between the user and the app. It is the responsibility of the user to input data that is accurate and consistent, whereas the app design must incorporate algorithms that can make accurate menstrual predictions. When comparing user preference of a paper pictorial blood assessment chart versus a mobile app to track menses, researchers determined that the most common reason that participants did not enter data in real-time on the mobile app was forgetting to enter the data.^4^ Thus, while the convenience of mobile apps could enhance user engagement and increase the opportunity for tracking anytime and anywhere, the use of apps could also increase the chance of irregular data reporting and even forgetting to report data until later, where recall biases might influence input accuracy.

In addition to accurate data entry, the various algorithms used in apps to arrive at menstrual predictions, such as the day of menstruation onset, day of ovulation, and most fertile days, rely on the innate assumptions of the algorithms. When comparing the accuracy of ovulation prediction between mobile apps and calendar methods, researchers reported that even though it is not possible to predict a fertile window that has a 100% chance of including the day of ovulation, the app predictions can improve over time with consistent and accurate tracking of menstrual-related variables.^9^ Thus, prediction improvements depend on the user inputting data in real time. An example of this is when experiencing cramps, a user would open the mobile app and record the date and severity of the cramps throughout the day. If the severity changes or another trackable variable appears, the user would log that data as well.

Real-time tracking has been reported to be fairly accurate in the symptom assessment of patients with irritable bowel syndrome (IBS).^10^ For menstrual tracking apps, an advantage over other tracking methods occurs with the ability of individuals to log the progression of symptoms that otherwise may not have been tracked through other methods. Progressive tracking gives insight into individual health status through the fluctuations in symptom occurrence and severity. This advantage was also noted in the IBS symptoms study.^10^

Improvements in an apps' menstrual prediction accuracy also depend on the adaptability of its algorithms. Do the algorithms account for changing menstrual cycle lengths? Cycle lengths can vary by 7 or more days in approximately 46% of individuals ages 18-40 years, and by 14 or more days in about 20% of individuals, which requires apps to account for this high prevalence of variability.^11^ Do the algorithms make predictions based on the typical 28-day cycle? Or can they be adjusted to the input of various cycle lengths over time? These questions should challenge menstrual cycle tracking app developers to collaborate with menstrual health experts to create apps that are accurate through continually adapting algorithms.

It is important to understand the limitations of a technology that can impact patient care and general wellness. Nurses should share that the algorithms used in these apps are not going to be 100% accurate, but they can be tailored toward the individual over time, so long as consistent, accurate, and real-time tracking of variables occurs.

## Navigating privacy and security concerns

In this digital age, the spaces, presence, and interactions of users on mobile devices have connected us to the world through communication and service exchanges. Oftentimes we do not think about the consequences of what is being done with our information when we exchange that data for a service, such as menstrual cycle tracking. The Health Insurance Portability and Accountability Act (HIPAA) is a 1996 federal law that protects our sensitive health information from misuse; however, when we input that information onto a health-focused app, HIPAA laws do not apply.^12^ Therefore, there is an increased risk of these apps using consumer data for numerous reasons without the patient's intentional knowledge and consent, including selling information to third parties for targeted marking and analytics.

The Federal Trade Commission reached a settlement in January 2020 with the menstrual tracking app Flo, after investigations reported that although Flo, “promised to keep users' health data private and only use it to provide the app's services to users” it instead, “disclosed health data from millions of users of its Flo Period & Ovulation Tracker app to third parties that provided marketing and analytics services to the app, including Facebook's analytics division, Google's analytics division, Google's Fabric service, AppsFlyer, and Flurry.”^13^ On the other hand, apps use consumer data to improve app design and/or function, others like Clue and Ovia, work with medical researchers to study women's health issues using anonymized information from these apps.^12^

App companies provide disclosure statements on what they do and do not do with consumer data, but these are oftentimes presented in lengthy, and jargon-filled language that most users just scroll past and mark as “read.” Researchers studied the readability and accessibility of Terms of Service and Privacy Policies of 15 menstrual tracking mobile apps and found that “on average, a college-level education was required to read both types of agreements.”^14^ According to the results of the Program for the International Assessment of Adult Competencies, 54% of adults between 16 and 74 years old read below the equivalent of a sixth-grade level.^15^ Considering this literacy level and that users of menstrual cycle tracking apps could be younger than 16, it is likely that a vast majority of users do not understand the contents of the agreements about how their data may be used and how it is shared within the app and possibly with third parties.

Until legislation can provide support toward consumer digital health data protection, there are steps consumers can take to better protect their information. As nurses, you can share the following advice with patients within their patient education package. There are various privacy control settings available both on apps and within mobile devices that users can modify to their needs. This is probably the best step to take in terms of privacy since you can opt out of permissions to share data with third parties and limit permissions on your location data and contacts.^12^ The use of a password manager can help keep passwords, PINs, and credit card numbers and security question answers safe and encrypted. Some examples are 1Password, Dashlane, KeePass, and LastPass.^12^ Although many menstrual tracking apps do not require a password to log in, users could implement two-factor authentication to further protect access to the app. Lastly, keep apps updated as frequent patches and fixes could be available to further protect your data.

**Figure:**
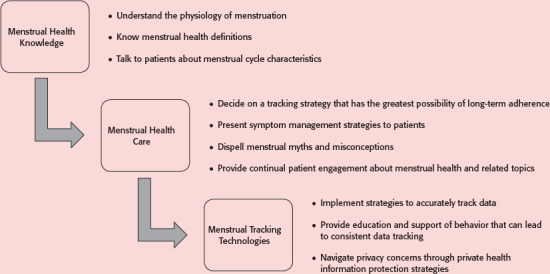
Menstrual healthcare plan using tracking technologies

## Nursing considerations

Using a digital, mobile way to track and understand menstrual-related variables is powerful in advancing menstrual health and empowering those who menstruate to reduce menstrual stigma and engage in active self-management. By understanding why individuals track their menstrual cycles and the benefits they can receive through tracking, nurses can design and implement menstrual care practices, such as recommending apps to use, and menstrual health information distribution strategies to better serve their patients while taking into regard the interconnectedness of menstrual health with social, economic, and political aspects of patients' lives.

For individual knowledge and action to be effective in combating menstrual stigma, nurses can initiate conversations about menstrual health, including normal characteristics of the menstrual cycle (such as, normal cycle ranges, duration of menses) and considerations for abnormalities like the severity of cramps and volume of blood loss. In addition, a discussion about how tracking menstrual variables can benefit patients' understanding of their bodies and how tracking information can help individualize their care can be incorporated into your nursing practice. For an illustration of a conceptual flow nurses can use to outline their menstrual cycle tracking, education, and patient-care approach using menses-related data from mobile tracking technologies, see *Menstrual health care plan using tracking technologies*.

Nurses can be menstrual health advocates in patient-care settings by discussing the benefits of tracking menstrual health variables in terms of self-awareness and health management along with educating patients about menstrual health using inclusive, compassionate language. Digital consumer education and steps to protect one's privacy in a digital space is an important component when advocating for use of a digital tracking method that involves sensitive health information. When it comes to specific menstrual cycle tracking apps, they need to be science-based similar to evidence-based nursing practice. Apps also need to be user-friendly, and users must understand the connection between accurate and meaningful menstrual predictions and having a detailed, consistent, and real-time data tracking behavior. Actively encouraging patients to use mobile tracking technology to log menstrual-related experiences can help combat menstrual stigma, promote greater self-awareness, and enhance their quality of life.
